# Combustion Synthesis of Magnetic Nanomaterials for Biomedical Applications

**DOI:** 10.3390/nano13131902

**Published:** 2023-06-21

**Authors:** Harutyun Gyulasaryan, Astghik Kuzanyan, Aram Manukyan, Alexander S. Mukasyan

**Affiliations:** 1 Institute for Physical Research, National Academy of Sciences of Armenia, Ashtarak-2, Ashtarak 0204, Armenia; gharut1989@gmail.com (H.G.); astghik.kuzanyan@gmail.com (A.K.); manukyan.ipr@gmail.com (A.M.); 2 Department of Chemical and Biomolecular Engineering, University of Notre Dame, Notre Dame, IN 46556, USA

**Keywords:** combustion synthesis, nanoparticles, magnetic properties, biomedical applications

## Abstract

Combustion synthesis is a green, energy-saving approach that permits an easy scale-up and continuous technologies. This process allows for synthesizing various nanoscale materials, including oxides, nitrides, sulfides, metals, and alloys. In this work, we critically review the reported results on the combustion synthesis of magnetic nanoparticles, focusing on their properties related to different bio-applications. We also analyze challenges and suggest specific directions of research, which lead to the improvement of the properties and stability of fabricated materials.

## 1. Introduction

Magnetic nanoparticles (MNPs) have gained significant attention in recent years, particularly in the biomedical field [1,2,3,4,5,6]. These nanoparticles exhibit high efficiency due to their large surface-area-to-volume ratio, chemical stability, and easy surface functionalization. The biocompatibility, rapid magnetic targeting, and high loading capacity of MNPs make them versatile in delivering therapeutic agents, improved multimodal imaging, and in magnetic hyperthermia [7,8,9,10,11,12,13,14,15,16,17,18,19]. In combination with other NPs, they also permit other bio-applications, such as serving as anti-microbial agents.

MNPs have demonstrated potential in targeting drug delivery to specific locations with the precise tracking of drug distribution, bio-distribution, and metabolism. These materials offer a promising strategy for site-specific drug delivery for cancerous tissues, inflammation, and cardiovascular diseases. The particles show excellent potential in controlling the treatment of the disease while minimizing toxicity to healthy cells. Furthermore, magnetic nanoparticles offer an efficient separation of biomolecules and cells. The magnetic properties of these nanoparticles allow them to immobilize biomolecules or cells on a magnetic surface [20,21,22,23,24,25]. MNPs can also be used for imaging by exploiting their magnetic properties, offering improved image contrast and resolution due to their magnetization behavior [26,27]. In hyperthermia for cancer treatment, the goal is to increase the temperature of the target tissue from 42° to 44°. This effect can be achieved using magnetic nanoparticles, known as magnetic hyperthermia [28,29]. The main challenges of this cancer therapy are improving the heating power of such nanoparticles and controlling the local temperature near the tumor. 

The synthesis of MNPs involves various methods, such as chemical precipitation, co-precipitation, sol–gel, microemulsion, hydrothermal, and combustion synthesis, to achieve the desired size, morphology, and magnetic properties [30,31]. The chemical precipitation method consists of precipitating metal ions from a salt solution by adding a reducing agent and a surfactant to control the particle size and morphology. Although this method produces MNPs with small size and narrow size distribution, it requires high-temperature conditions and toxic reagents [32,33,34,35]. In the co-precipitation method by changing the pH and reaction time, the size and magnetic properties of the MNPs can be controlled and produced at a lower cost with good magnetic properties [36,37,38]. The sol–gel method includes the hydrolysis and condensation of metal alkoxides in a sol–gel matrix [39,40,41,42,43,44]. This method produces large quantities of nanoparticles with good magnetic properties but is time-consuming and requires high-temperature conditions. 

The micro-emulsion comprises systems consisting of two immiscible phases (e.g., oil and water) and a surfactant [45,46,47,48,49]. Metal ions are reduced in the nanoscale droplets, resulting in MNPs with a narrow particle size distribution. This process is relatively simple, fast, and produces MNPs with a narrow particle size distribution. Finally, the hydrothermal method involves reacting metal salts in an autoclave under high temperature and pressure to create MNPs [50,51,52,53]. By modifying the reaction time, temperature, and precursor concentration, the size and shape of the nanoparticles can be controlled. Since this technique involves high-temperature conditions, it yields MNPs with excellent crystallinity.

Solution combustion synthesis (SCS) is a green, energy-saving approach that allows for an easy scale-up and continuous technologies [54]. SCS contains self-sustained exothermic reactions along an aqueous or sol−gel media. This process allows for synthesizing various nanoscale materials, including oxides, nitrides, sulfides, metals, and alloys. 

In this work, we critically review the reported results on the SCS of magnetic nanoparticles, focusing on their properties related to different bio-applications. We also analyze challenges and suggest specific directions of research, which will allow the improvement of the properties and stability of MNPs fabricated by the SCS method.

## 2. Solution Combustion Synthesis: Fundamentals

The solution combustion synthesis (SCS) method is a highly versatile approach and relies on the occurrence of non-catalytic, self-sustained exothermic reactions in solutions or gels [55]. Such reactive solutions contain oxidizers and fuels dissolved in a solvent, and the precursors used can be seen in Table 1.

**Figure 1:**
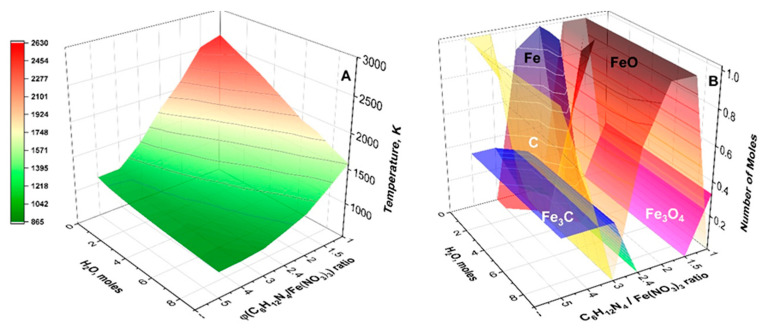
Calculated adiabatic temperature (**A**) and equilibrium solid products (**B**) for Fe(NO_3_)_3_-C_6_H_12_N_4_-H_2_O system vs. fuel/oxidizer molar ratio (ϕ) and amount of water (n). (Reprinted with permission from [56]. Copyright 2019 American Chemical Society). The above features delineate the versatility of the SCS approach.

For instance, in the iron-nitrate-hydrates–HMTA system in an argon atmosphere, a self-sustained reaction in aqueous solution of the metal nitrite and the fuel, Fe(NO_3_)_3_ + nH_2_O + C_6_H_12_N_4_, results in the formation of various magnetic phases [56]. The reaction is highly exothermic, and the adiabatic combustion temperature (T_ad_) exceeds 2500 K. The T_ad_ can be controlled by adjusting the amount of water (n) and fuel-to-oxidizer molar ratio ϕ = C_6_H_12_N_4_/Fe(NO_3_)_3_. It can be seen (Figure 1A) that at ϕ = 1 and n = 0, T_ad_ = 2630 K, while at n = 4 and ϕ = 4, it is below 1000 K. The phase composition of the solid-state product changes correspondingly (Figure 1B). The wide specter of magnetic phases, including Fe, Fe_3_O_4_, Fe_3_C, and Fe_3_N, can be synthesized in the same system by optimizing combustion parameters (n, ϕ). Close to stoichiometry (ϕ = 1), the Fe_3_O_4_ phase is in equilibrium, while at large ϕ, the Fe_3_C prevails. The narrow parametric n-ϕ region corresponds to the formation of the pure iron phase.

**Figure 2:**
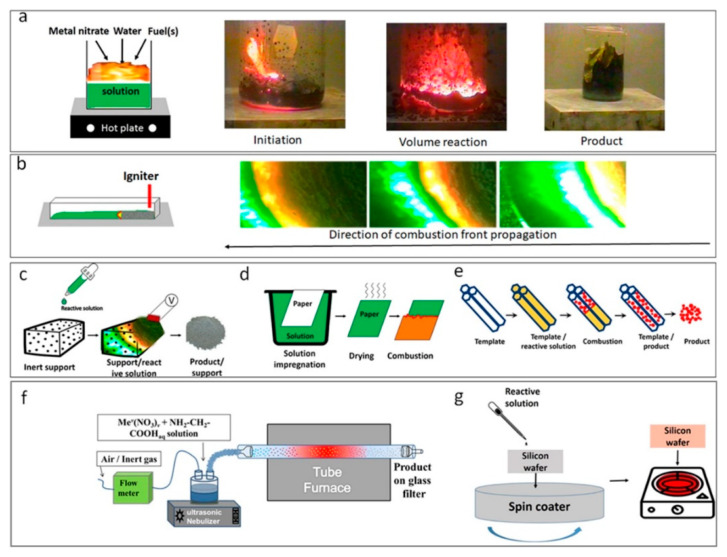
Different SCS modes: (**a**) volume combustion synthesis; (**b**) self-propagating high-temperature synthesis; (**c**) impregnated SCS; (**d**) cellulose-assisted SCS; (**e**) templated SCS; (**f**) spray SCS; (**g**) SCS of thin films. (Reprinted with permission from [57]. Copyright 2020 Elsevier).

SCS can be achieved through different reaction modes [55,57], including volume SCS, self-propagating high-temperature synthesis (SHS), impregnated SCS, cellulose-assisted SCS, templated SCS, spray SCS, and SCS of thin films (Figure 2). Each of these modes has unique features and can be applied to fabricate various materials, including oxides, metals, alloys, nitrides, and carbides, with high specific surface areas and narrow size distributions [54,55].

**Figure 3:**
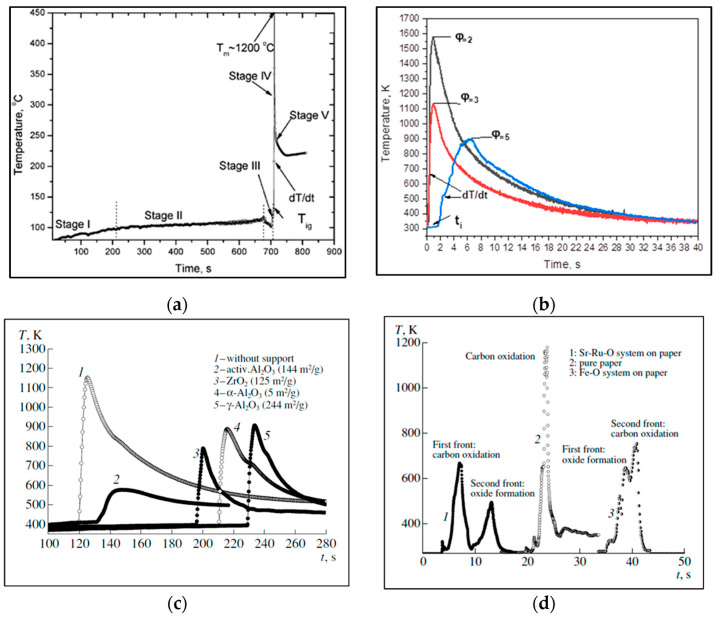
Typical temperature–time profiles for different SCS modes: (**a**) VSCS; (**b**) SHS; (**c**) impregnated SCS—inert substrate; (**d**) cellulose-assisted SCS. (Reprinted with permission from [58]. Copyright 2004 American Chemical Society; Reprinted with permission from [59]. Copyright 2020 Springer Nature; Reprinted with permission from [60]. Copyright 2005 American Chemical Society; Reprinted with permission from [62]. Copyright 2007 John Wiley and Sons).

Among the different SCS modes, volume SCS (VSCS) is widely used (Figure 2a). In this case, a solution is uniformly preheated to the self-ignition temperature, which takes place essentially along the entire reactive volume. An example of a time–temperature profile for VSCS in the Fe(NO_3_)_3_–glycine system (ϕ = 1) is shown in Figure 3a [58]. It can be seen (Figure 2b) that the duration of the high-temperature stage IV is extremely short. In the case of the SHS mode, a solution is locally preheated to initiate a reaction and the high-temperature reaction front propagates along the media. The width of the high-temperature reaction zone is more comprehensive as compared to that of the VSCS (Figure 3b) [59].

The liquid state of the reaction media permits different types of impregnated SCS modes. The solution can be impregnated into the inert porous media, followed by reaction initiation (Figure 2c). In this case, the maximum reaction temperature is lower as compared to the SHS mode because of the inert dilution of the system (Figure 3c; Ref. [60]). This approach was used to prepare high-specific-surface-area-supported catalysts [61]. The solution can also be impregnated into active porous media, e.g., cellulose [62,63]. Cellulose-impregnated SCS is typically used for low-exothermic systems because the combustion of cellulose contributes to the total exothermicity of the system. For different systems, the burning of cellulose may precede or follow the reaction in the solution (Figure 3d; Refs. [62,64]). The catalytic or non-catalytic high-specific-surface-area materials can be prepared by using these modes [60,61,62,63,64].

The last version of the impregnated mode is templated-SCS (Figure 2e). In this case, a solution is impregnated into the media with the desired pore size distribution, e.g., silica nanotubes. After a self-sustained reaction, the template is removed by dissolution in an appropriate solvent, while synthesized particles have a narrow size distribution corresponding to the nanotubes’ diameter [65]. 

The other SCS mode is spray solution combustion, which combines concepts of SCS and aerosol spray pyrolysis (Figure 2f). In this case, the aqueous reaction solution was placed in the chamber of the inhaler and sprayed into a tubular furnace by a carrier gas (argon, nitrogen, air). The initial liquid spheres combusted and converted to the ideal solid, hollow spheres with diameters controlled by the diameter of the initial droplet and wall thickness in the range of 10–20 nm [58]. Finally, the SCS can be used for the fabrication of thin coatings (Figure 2g). The reactive solution is spin-coated onto the desired substrate, followed by reaction initiation in the layer [66,67].

Overall, the SCS method is highly versatile and can be applied to synthesize different magnetic phases and materials with unique properties [54,55]. Further investigations are needed to optimize the parameters and reaction modes to yield the desired properties. Below, we overview reports on SCS of the nanoparticles, focusing on their magnetic properties and biomedical applications. 

## 3. Solution Combustion Synthesis of Magnetic Compounds

Various magnetic nanoparticles were created using the SCS method. Below, we focus on the most extensively studied types, such as iron- and nickel-based materials, as well as complex oxides. Moreover, we provide a brief overview of the SCS process for zinc- and calcium-based nanoparticles, which, when paired with magnetic NPs, demonstrate potential for a diverse array of biomedical applications.

### 3.1. Iron-Oxide-Based Materials

In recent work, the iron oxide magnetic particles were fabricated by SCS using citric acid as a fuel [58]. The aqueous solution of ferric nitrate nonahydrate, Fe(NO_3_)_3_·9H_2_O, and citric acid, C_6_H_8_O_7_, mixed in a molar ratio of 6:5 (ϕ = 0.8) was utilized. The reaction can be represented as follows:

(1) $$Fe{\left(NO_{3}\right)}_{3}+\left(\phi -1/6\right)C_{6}H_{8}O_{7}+\left[9/2\left(\phi -1\right)\right])O_{2}\to 1/2Fe_{2}O_{3}+\left[6\left(\phi -1/6\right)\right]CO_{2}{↑}_{g}+\left[4\left(\phi -1/6\right)\right]H_{2}O+3/2N_{2}{↑}_{g}$$

Heating sources such as a heating mantle with a temperature of 450 °C and a microwave oven (245 GHz, 800 W) were used in the VSCS process, but exact synthesis conditions were not reported. We understand the complications associated with temperature measurements during volume SCS, but it is a critical issue for controlling the microstructure and, thus, properties of the obtained materials.

The XRD analysis revealed that the phase composition of the produced powders was influenced by the heating method used. In the case of heating in the mantle, the product (P1) involved primarily (97%) magnetite (Fe_3_O_4_), while in the case of the microwave heating, the product (P2) was primarily (85%) maghemite (γ-Fe_2_O_3_) (Table 2). 

**Figure 4:**
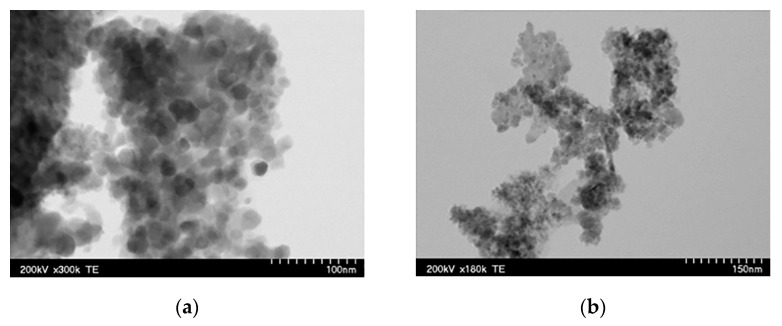
TEM images of powders synthesized by VSCS: (**a**) mantle heating; (**b**) microwave. (Reprinted with permission from [68]. Copyright 2021 Elsevier).

The TEM images of both products are shown in Figure 4. The medium particle size was 15 and 11 nm for P1 and P2, respectively. Correspondingly, the BET-specific surface area was 42 and 71 m^2^/g, respectively. Both powders displayed superparamagnetic behavior with a saturation magnetization of 67(P1) and 42(P2) emu/g, respectively.

**Figure 5:**
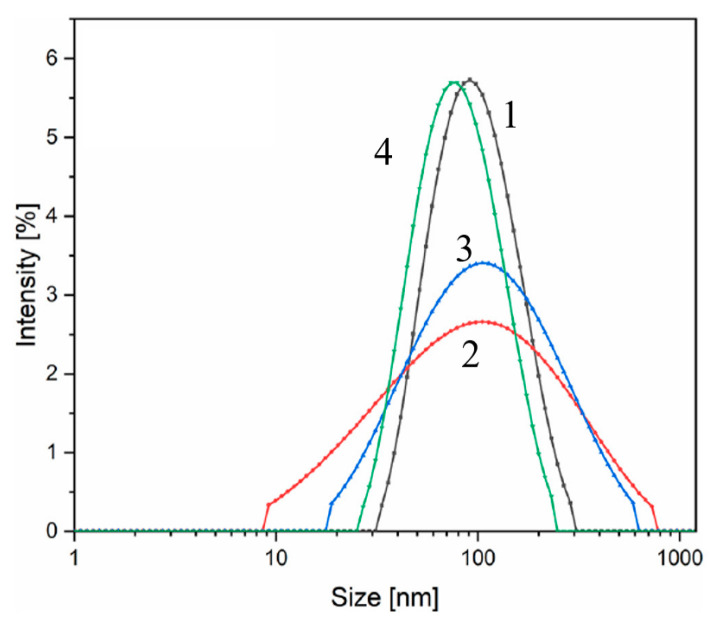
The intensity distribution of particles size (DLS) of different colloidal suspensions (O—oleic acid; W—distilled water; T—Tween 80; PBS—phosphate-buffered saline). (Reprinted with permission from [68]. Copyright 2021 Elsevier).

Based on the structural characteristics, P2 particles were selected for the preparation of colloidal solutions by using different surfactants and solvents. Figure 5 represents the results recorded for four colloidal suspensions in terms of their particle size distribution. It can be seen that all the colloidal suspensions possess a unimodal particle size distribution with an average hydrodynamic diameter of 90 nm (O/W—line 1), 105 nm (O/PBS—line 2 and T/W—line 3), and 80 nm (T/PBS—line 4).

The characteristics of the colloidal solution are presented in Table 3. Pure oleic acid provides a higher absolute value for negative zeta potential, indicating a higher stability of the colloidal suspension. In addition, the lower polydispersity index (PDI) suggests better homogeneity of the media. The work demonstrates that SCS is a versatile method for the synthesis of iron oxide nanoparticles of about 10 nm, which allows the fabrication of stable colloidal suspensions.

The aqueous solution of ferric nitrate nonahydrate, Fe(NO_3_)_3_·9H_2_O, and citric acid, C_6_H_8_O_7_ but with molar ratio ϕ = 3, was used to fabricate α-Fe_2_O_3_ particles [69]. The particles were produced in volume SCS mode, placing the solution on the hot plate preheated to 500 °C. Again, the time–temperature of the process was not reported. A well-crystalline rhombohedral α-Fe_2_O_3_ phase with a crystallinity size of ~27 nm was obtained. The particles possessed a spherical morphology with an average diameter of ~75 nm. These particles were also mixed with thin 2D-carbon-flakes having a size of ~2 µm. 

The cellular toxicity of α-Fe_2_O_3_, carbon nano-plates, and α-Fe_2_O_3_/C nanocomposites was studied using MTT assay and nuclear imaging based on the cell morphological changes on both human lung cancer cell line A549 and a non-cancerous cell line. The relative cell viability (%) was estimated. The results demonstrated that the pure and composite material exhibited a viability above 70% on non-cancerous cell lines and an inhibition of around 60% on A549 lung cancer, indicating that the α-Fe_2_O_3_/C nanocomposite is biocompatible and can be used for biological applications and anticancer therapy.

**Figure 6:**
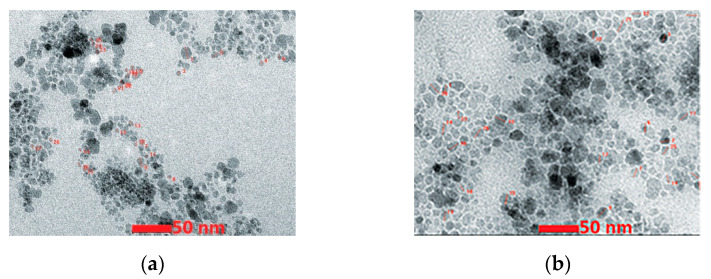
TEM images of (**a**) Fe_3_O_4_ and (**b**) γ-F_2_O_3_ particles (adopted from [70]).

The reagents used for the SCS of Fe_3_O_4_ (magnetite) and γ-Fe_2_O_3_ (maghemite) nanoparticles and their colloidal suspensions were: Fe(NO_3_)_3_·9H_2_O as an oxidizing agent, and citric acid and glycose D-(+)-C_6_H_12_O_6_ as fuels [70]. The synthesis procedure was not described in detail. Using citric acid as a fuel led to the formation of the magnetite phase, while glucose resulted in the synthesis of γ-Fe_2_O_3_ particles. It is worth noting that the latter contradicts the work results from which the synthesis protocol was utilized, where both fuels led to the formation of Fe_3_O_4_. The TEM images of the Fe_3_O_4_ and γ-Fe_2_O_3_ particles are shown in Figure 6 and the powder’s characteristics are presented in Table 4.

The authors were able to create a stable colloidal suspension from the particles by using oleic acid as a surfactant and water and PBS as solvent media. It was more important to test their effects in vitro on human keratinocytes and in vivo by employing an animal model of acute dermal toxicity. They found a lack of toxicity for normal human cells induced by the iron oxide nanoparticles colloidal suspensions after an exposure of 24 h. The dermal acute toxicity test showed that the applications of the colloidal suspensions on female and male SKH-1 hairless mice were not associated with significant changes in the quality of skin function. The above works prove that 10–70 nm spherical biocompatible nanoparticles of different iron oxides can be fabricated by SCS.

**Figure 7:**
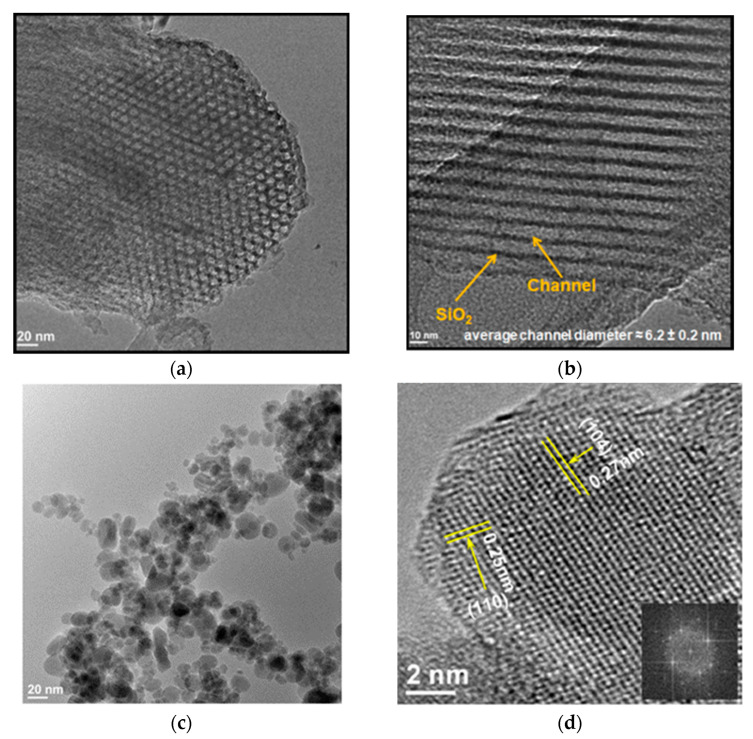
TEM images of (**a**,**b**) SBA template and (**c**,**d**) Fe_2_O_3_ nanoparticles synthesized in SHS mode. (Reprinted with permission from [65]. Copyright 2014 American Chemical Society).

The mesoporous silica (SBA-15) was used as a template (Figure 7a,b) to fabricate nanoparticles below 5 nm in size [65]. The iron-nonahydrate–glycine–ammonium-nitrate aqueous solution was impregnated into the porous high-surface-area-BET (~780 m^2^/g) template, which involves channels with a diameter ~6 nm ±0.2 nm. The TEM image (Figure 7c) of combustion products after leaching the template indicates that ~95% of hematite particles were smaller than 5 nm, with an average size of ~3.5 nm. The high-resolution TEM (HRTEM) image (Figure 7d) provides information on the atomic structure and morphology of α-Fe_2_O_3_ nanoparticles. The interplanar spacing of 0.27 nm corresponds to the d104 spacing between (104) atomic planes of the crystal structure. It was shown that because of the ultralow sizes and high crystallinity, the combustion-derived α-Fe_2_O_3_ nanoparticles exhibited superparamagnetic properties in the temperature range of 70–300 K. The high specific surface area (132 m^2^/g) of the powder indicates the vital role of surface magnetic spins, resulting in high magnetization at 300 K.

The results above demonstrate that iron oxide NPs with the desired phase composition can be successfully fabricated by SCS in the range size from 4 to 100 nm. The obtained materials were shown to be biocompatible and allowed the preparation of a stable colloidal suspension. The following research stage should involve extensive in vivo and in vitro studies of such MNPs for different biomedical applications.

### 3.2. Nickel Oxide and Ni-Based Alloys 

**Figure 8:**
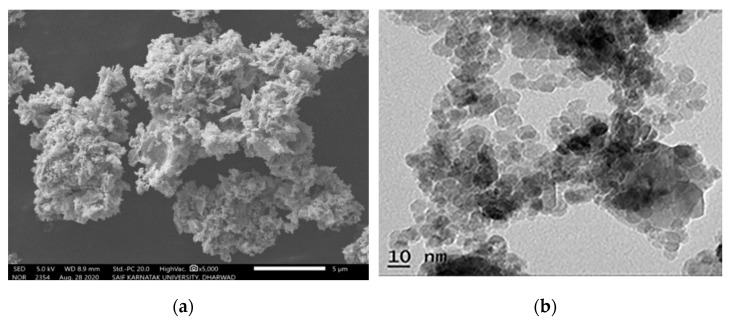
SEM (**a**) and TEM (**b**) images of NiO particles (adopted from [73]).

The SCS process of nickel oxide (NiO) is a process that has been widely used to investigate the intrinsic mechanism of self-sustained chemical reactions in homogeneous solutions (cf., [71,72]). Several fuels have been utilized, as shown in Table 1, to fabricate NiO nanoparticles. The first system to produce pure Ni in the combustion wave was the nickel-nitrite–glycine system [71]. However, a recent publication suggested the exotic fuel, i.e., Areca catechu leaf extract, with the following elemental composition: carbon (55 wt%), oxygen (36 wt.%), potassium (4 wt.%), chlorine (2.5 wt%), silicon (1 wt%), and calcium (0.7 wt%)) [73]. The SCS was accomplished in VSCS mode with a furnace temperature of 500 °C. The well-crystalline FCC NiO phase possesses a crystalline size of ~5.6 nm. The SEM and TEM images of the particles are shown in Figure 8, indicating that the powder agglomerates involve particles with a size of ~10 nm.

Furthermore, the synthesized NiO NPs’ effectiveness in inhibiting pancreatic α-amylase and their anticancer activity was compared with the standard drug Metformin. Metformin showed inhibitory effects on α-amylase activity with a concentration of test drug needed to inhibit cell growth by 50% (IC_50_) of 232 µg/mL. The IC_50_ value of the extract was found to be 268 µg/mL. The biological assay results revealed that NiO NPs exhibited significant cytotoxicity against human lung cancer cells as well, showing considerable cell viability.

**Figure 9:**
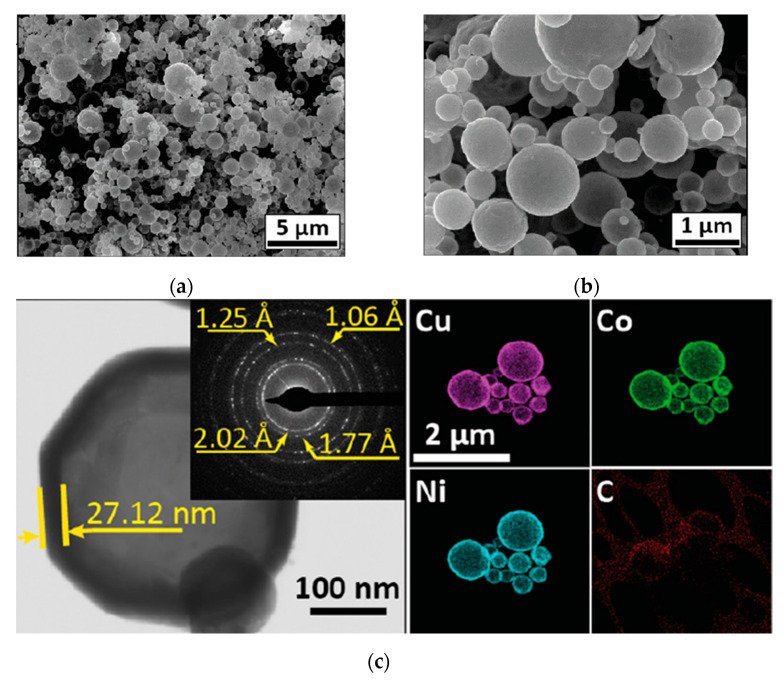
SEM (**a**,**b**) and TEM (**c**) images with ED and EDX diagnostics. (Reprinted with permission from [74]. Copyright 2021 Elsevier).

In another recent publication [74], the authors report the SCS of CoCuNi alloy, which possesses the morphology of hollow spheres (Figure 9), using the spray SCS mode to produce this unique structure. The CoCuNi shell’s thickness ranged from 10 to 30 nm, and the outer diameter of the spheres was in the range of 0.2 to 2 µm. The magnetic properties of the alloy were studied at room temperature, revealing high saturation magnetization in the range of 65–75 emu/g, making it an excellent candidate for drug delivery applications.

In conclusion, Ni-based nano-structured materials can be synthesized using different SCS modes. Through understanding the reaction mechanism, the morphology of the particles obtained can be controlled, thus controlling their physical and chemical properties required for different bio-applications.

### 3.3. Complex Oxides

The Solution Combustion Synthesis (SCS) technique is an effective method for producing complex oxides that possess desirable magnetic properties. One example of this is the synthesis of sub-micrometric (250–300 nm) BiFeO_3_ particles using the volume combustion synthesized mode [75]. Bismuth nitrate pentahydrate, iron nitrate nonahydrate, nitric acid, and glycine were used as precursors in this work. The balanced chemical reaction can be written as follows:

(2) $$\begin{array}{r} Bi{\left(NO_{3}\right)}_{3}+Fe{\left(NO_{3}\right)}_{3}+2pHNO_{3}+4nH_{2}NCH_{2}COOH+\left[9n-2.5p-7.5\right]O_{2}\to \\ \to \;BiFeO_{3}\left(s\right)+\left(10n+p\right)\cdot H_{2}O\left(g\right)+8nCO_{2}\left(g\right)+\left(2n+p+3\right)N_{2}\left(g\right) \end{array}$$

The n and p coefficients represent the number of glycine and nitric acid moles used per mole of Bi(NO_3_)_3_·5H_2_O, respectively. It is worth noting that all synthesized powders were post-annealed first at 350 °C for 2 h and then at 500 °C for 2 h. Finally, the powders were heat-treated at 600 °C for 3 h in air. 

**Figure 10:**
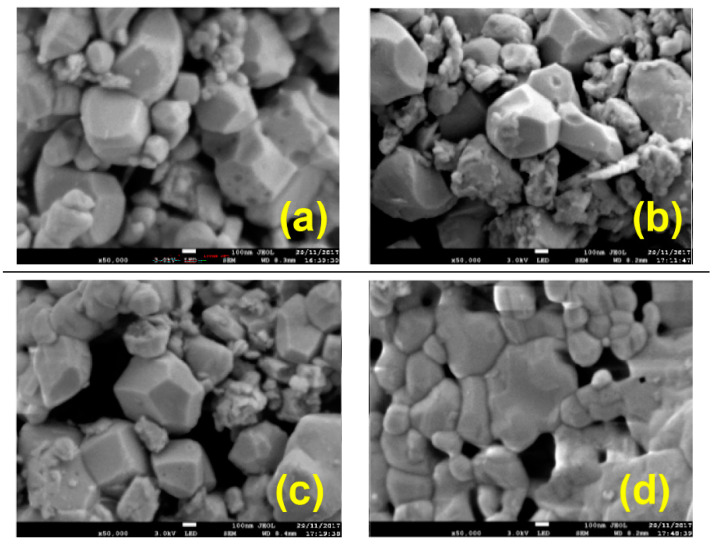
Typical SEM images of BiFeO_3_ samples synthesized using four oxidizing/reducing agent ratios: 10 (**a**), 7.4 (**b**), 5.2 (**c**), and 3.6 (**d**); the length of the scale bar is 100 nm. (Reprinted with permission from [75] Copyright 2018 American Chemical Society).

The XRD analysis of all powders indicates the formation of a rhombohedral BiFeO_3_ phase with a size of the crystallite range of 35 to 60 nm. Figure 10 shows the typical microstructures of the powders after annealing at 600 °C. The materials involved well-faceted crystals in the size range of a few nanometers to a few hundred nanometers. It can be seen that BiFeO_3_ powders synthesized with a high-oxidizing/reducing-agent ratio (10, 7.4, and 5.2; Figure 10a–c) possessed a wider particle size distribution, as compared to that fabricated with a ratio of 3.6 (Figure 10d). 

**Figure 11:**
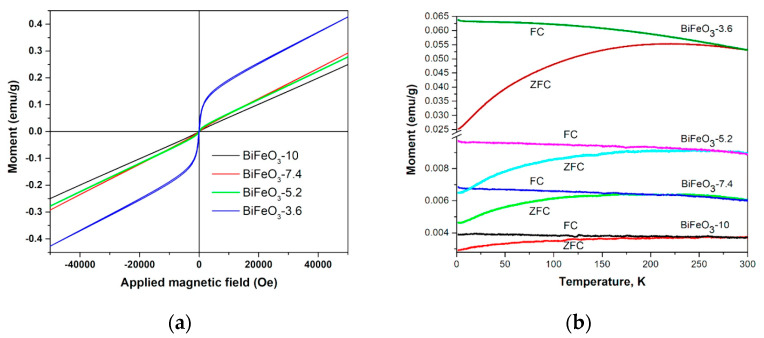
(**a**) Magnetization versus applied magnetic field curves at 300 K and (**b**) zero-field-cooled (ZFC) and field-cooled (FC) magnetization curves (under 500 Oe applied magnetic field) for the BiFeO_3_ samples. (Reprinted with permission from [75] Copyright 2018 American Chemical Society).

Figure 11 shows the room-temperature magnetization curves along with the zero-field-cooled (ZFC) and field-cooled (FC) magnetization of the different BiFeO_3_ powders. The shape of the magnetization curves for the samples with the high-oxidizing/reducing agent ratio exhibited characteristics of an antiferromagnetic material. In contrast, the magnetization curve of the BiFeO_3_-3.6 sample displayed a combination of antiferromagnetic and superparamagnetic features. The existence of ferromagnetic ordering in all powders was confirmed by estimating the coercive field at different temperatures, as presented in Table 5. The coercivity of all the samples increased with the decrease in temperature, which occurred in ferromagnetic materials. 

The BiFeO_3_–3.6 particles had a room-temperature magnetic moment almost twice those of other fabricated materials (Figure 11a). The microstructure of the particles explained this effect. Specifically, the BiFeO_3_-3.6 sample consisted of well-crystalline submicron particles of ∼100 nm size, which favors the increase in magnetization by uncompensated spins at the surface or grain boundaries.

It was concluded that by varying the ratios of NO3- ions and glycine, it was possible to avoid the formation of typical impurity phases such as Bi_2_Fe_4_O_9_ and significantly improve the magnetic properties. Moreover, the BiFeO_3_ particles manifested higher splitting temperatures between their ZFC and FC magnetization curves and moderate coercive field, which led to their stable antiferromagnetic behavior over a wide temperature range (Figure 11b).

**Figure 12:**
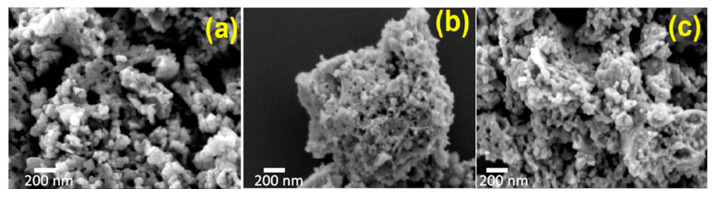
SEM images of the (**a**) CoFe_2_O_4_, (**b**) NiFe_2_O_4_, and (**c**) Co_0.5_Ni_0.5_Fe_2_O_4_ nanoparticles. (Reprinted with permission from [76]. Copyright 2018 American Chemical Society).

Another example is the SCS of nano-scale ferrites, where CoFe_2_O_4_, NiFe_2_O_4_, and Co_0.5_Ni_0.5_Fe_2_O_4_ ferrite NPs were produced using cobalt, nickel, and iron nitrates precursors as the oxidizing agent and glycine as the fuel [76]. The typical microstructures of the synthesized powders are shown in Figure 12. It was estimated that the average size of the particles in the CoFe_2_O_4_, NiFe_2_O_4_, and Co_0.5_Ni _0.5_Fe_2_O_4_ samples were 40 ± 10 nm, 26 ± 8 nm, and 32 ± 7 nm, respectively.

**Figure 13:**
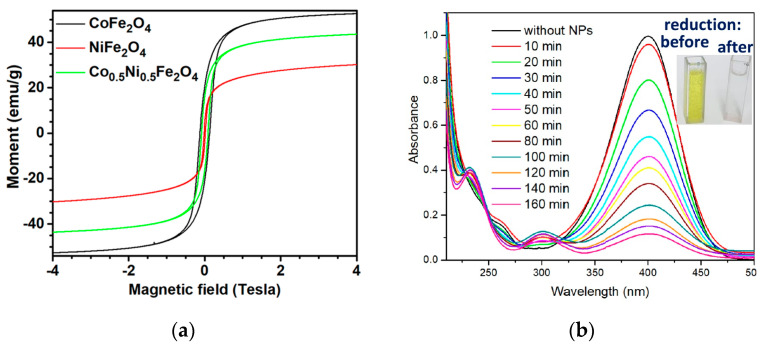
(**a**) Magnetization vs. applied magnetic field curves at 300 K for CoFe_2_O_4_-6, NiFe_2_O_4_-6, and Co_0.5_Ni_0.5_Fe_2_O_4_-6; (**b**) UV−vis absorption spectra correspond to the progressive reduction of 4-nitrophenol to 4-aminophenol using the NiFe_2_O_4_-3 sample as the catalyst (**b**). (Reprinted with permission from [76]. Copyright 2018 American Chemical Society).

Magnetization curves of the CoFe_2_O_4_, NiFe_2_O_4_, and Co_0.5_Ni_0.5_Fe_2_O_4_ nanoparticles were investigated at different temperatures. The hysteresis loops of CoFe_2_O_4_ NPs showed typical characteristics of hard ferrimagnetic material, whereas the magnetization curves of NiFe_2_O_4_ NPs corresponded to soft ferromagnetic material (Figure 13a). The room-temperature saturation magnetizations (M_s_) of the CoFe_2_O_4_, NiFe_2_O_4_, and Co_0.5_Ni_0.5_Fe_2_O_4_ were 53, 30, and 44 emu/g, respectively. The magnetic coercivity (H_c_) of NiFe_2_O_4_ NPs was only 159 Oe at room temperature, but it significantly increased to 886 Oe when half of the Ni^2+^ cations were replaced by Co^2+^ cations, resulting in Co_0.5_Ni_0.5_Fe_2_O_4_ nanoparticles. 

The effects of phase purity and stoichiometry on the catalytic behavior of the metal ferrites were studied by evaluating their reduction efficiency of 4-nitrophenol, a common organic contaminant in wastewater. The phase-impure NiFe_2_O_4_ sample, which featured segregated metallic Ni clusters on its surface, exhibited exceptional performance in the reduction of 4-nitrophenol (Figure 13b).

In addition, SCS was utilized for synthesizing Co_0.5_Me_0.5_Fe_2_O_4_ (Me = Mn and Zn) ferrites [77]. These ferrites were synthesized by using corresponding metal nitrites as oxidizers and oxalyl dihydrazide (ODH: C_2_H_6_N_4_O_2_) as a fuel. The volume reaction mode was used with a furnace temperature of 400 °C. It is worth noting that no additional calcination stages were applied. The obtained ferrites were well-crystalline and had a particle size distribution ranging from 25 nm to 30 nm. The magnetic hysteresis loops confirmed ferromagnetic behavior, where Co _0.5_Zn _0.5_Fe_2_O_4_ exhibited the highest saturation magnetization value (76 emu/g). Dielectric measurements showed that doping the parent CoFe_2_O_4_ nano-ferrite by Mn^2+^, Ni^2+^, or Zn^2+^ led to a decrease in the dielectric constant values.

Therefore, SCS is a powerful method for synthesizing complex oxides and producing various nanomaterials with attractive magnetic properties that can be optimized for different applications. The SCS also permits the fabrication of various nanomaterials that, in combination with magnetic phases, are suitable for different bio-applications. Let us briefly overview the most widely investigated systems. 

### 3.4. Zinc Oxide

Zinc oxide (ZnO) is a safe inorganic antimicrobial agent recognized by the US FDA for use in humans. The available literature suggests that ZnO particles of varying morphology and size can be produced using SCS [78,79,80,81,82,83,84,85]. Synthesis typically involves using zinc nitrate hexahydrate with different fuels. Let us overview some examples.

Zinc oxide nanomaterials doped with Ag and Au were synthesized by using urea as a fuel, and silver nitrate (AgNO_3_, 98.9%) and tetra chloroauric-III-acid hydrate (HAuCl_4_·xH_2_O, 98%) for doping [81]. The VSCS mode was used with a furnace temperature of 500 °C. The result was sub-micron (100–500 nm) flower-like 3D particles with uniformly distributed Au and Ag on the surface, with an average cluster size of 3–10 nm. 

The antimicrobial study of such NPs was performed on Gram-negative *Escherichia coli* and Gram-positive *Staphylococcus aureus*, while antifungal tests were carried out with the yeast *Eremothecium ashbyii*. The results showed that the Ag-doped ZnO nanomaterial had a degradation efficiency of 45% against methylene blue. Antimicrobial and antifungal activity studies have shown that pure ZnO is more effective against *Escherichia coli* and *Staphylococcus aureus*, and Ag-doped ZnO is more effective against *Eremothecium ashbyii*.

**Figure 14:**
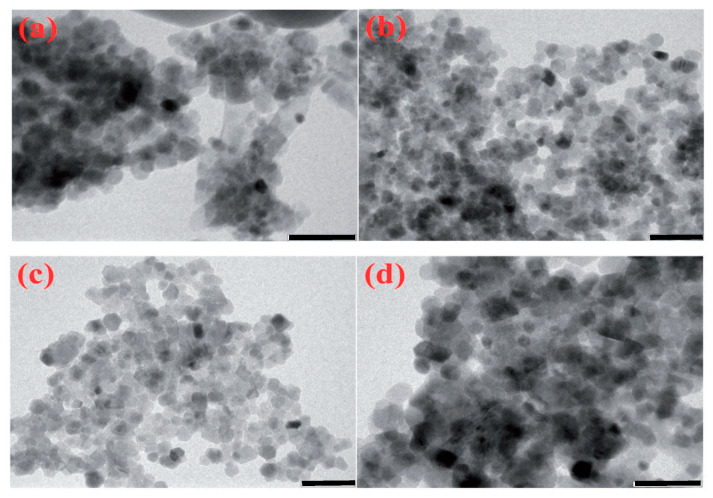
TEM images of the ZnO particles obtained with different fuels: (**a**) lactose; (**b**) A. indicum; (**c**) M. azedarch; (**d**) I. tinctoria; scale bars are 50 nm for all images (Reprinted with permission from [82]. Copyright 2018 Taylor & Francis).

Other fuels were used to fabricate ZnO nanoparticles, such as aqueous leaf extracts of Abutilon indicum, Melia azedarach, Indigofera tinctoria, and lactose monohydrate [82]. The furnace temperature was 375 ± 10 °C. XRD analysis indicated high crystallinity of the fabricated ZnO phase. TEM images of different particles are presented in Figure 14, and some parameters extracted from XRD and BET measurements are shown in Table 6. The average particle size of ZnO (lactose), ZnO (Abutilon indicum), ZnO (Melia azedarach), and ZnO (Indigofera tinctoria) was found to be 26, 15, 12, and 21 nm, respectively.

This effect is probably related to the higher surface area of these particles (see Table 6. In addition, the results of cytotoxicity studies by hemolysis assay suggest that all ZnO NPs fabricated by SCS are biocompatible at their lower concentration, up to 2.5 mg/mL. 

The MTT assay in DU-145 and Calu-6 cells showed that all ZnO NPs induced a dose-dependent toxicity in both cells, with materials using aqueous leaf extracts showing a better activity than those synthesized with lactose as fuel. ZnO (M) synthesized with the aqueous extract of leaves of M. azedarach showed a higher anticancer activity in both DU-145 and Calu-6 cells. This effect is related to the higher surface area of these particles (Table 6).

The above examples show that SCS allows the synthesis of ZnO nanoparticles in a wide range (12–500 nm) of sizes. It also allows easy doping of such particles with the desired elements (see also [83,84,85]).

### 3.5. Calcium-Based Compounds

Calcium phosphates (CaPs) are ceramics that are being explored for biomedical and orthopedic applications [86]. However, their poor biodegradability, such as in hydroxyapatite, remains a challenge. Two approaches to enhance the ceramics’ properties are to (i) dope and (ii) use a new synthesis method [87]. The SCS method, in particular, is an attractive pathway for doping.

**Figure 15:**
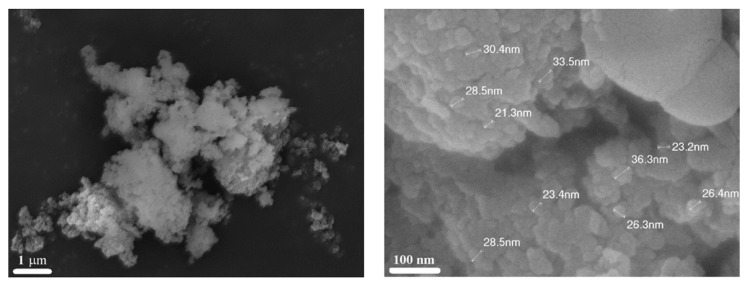
SEM images the CaP powder obtained by SCS and heat-treated at 800 °C for 2 h. It can be seen that the aggregates of about 5 μm in size involve nanoparticles of spherical morphology and average grain size 43 ± 1 nm. (Reprinted with permission from [88]. Copyright 2018 Elsevier).

For example, the CaP nanostructures were synthesized using calcium nitrate tetrahydrate Ca(NO_3_)_2_∙4H_2_O, di-ammonium hydrogen phosphate (NH_4_)_2_∙HPO_4_, glycine, and nitric acid [88]. The reactive aqueous solution was heated to a temperature of 60 °C for total evaporation of the solvent. Subsequently, the temperature was increased to 140 °C until the initiation of the combustion reaction. The obtained ash was calcined at 800 °C for 2 h. XRD analysis revealed the formation of hydroxyapatite and beta-tricalcium phosphate phases, with traces of calcium pyrophosphate. The later phase disappeared after calcination. The typical microstructures of the powder are shown in Figure 15.

The synthesized CaP powder was tested as a potential support for photoactive drugs using hypericin (HY). It was demonstrated that 54 μg/mL is the amount of CaP particles loaded with HY in the presence of light needed for reducing the parasites’ population by half. This value is low compared to the amount of free HY (about 458 μg/mL) needed to exhibit an EC_50_ effect.

Si-doped CaP ceramics were also fabricated using calcium nitrate tetrahydrate (Ca(NO_3_)_2_·4H_2_O), ammonium dihydrogen phosphate (NH_4_)(H_2_PO_4_), and tetraethyl orthosilicate (TEOS) (Si(OC_2_H_5_)_4_) as precursors [89]. The glycine and citric acid were employed as fuels. The TEOS was hydrolyzed in the presence of HNO_3_ and deionized water. The solutions were sequentially supplemented with calcium nitrate, ammonium dihydrogen phosphate, and fuel and heated up to about 330°C using a digital hot plate. XRD patterns showed the presence of primary hydroxyapatite (HA) and some amount of beta-tricalcium phosphate (βTCP) in all synthesized samples (Table 7). Crystallite sizes of HA and βTCP in the powders synthesized using glycine and citric acid were 15 nm and 43 nm, respectively. 

**Figure 16:**
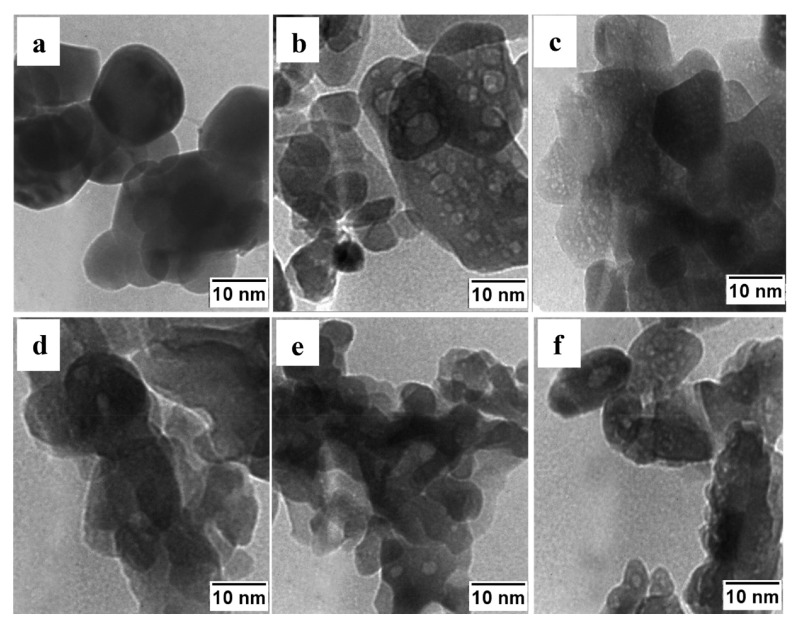
TEM micrographs of the powders synthesized using glycine (undoped (**a**), and doped with 0.1 (**b**) and 0.4 (**c**) mole Si^4+^ ions) and citric acid (undoped (**d**), and doped with 0.1 (**e**) and 0.4 mol (**f**) Si^4+^ ions). (Reprinted with permission from [89]. Copyright 2020 Elsevier).

The specific surface areas (SSAs) for the undoped powders synthesized using glycine and citric acid were 38 and 20 m^2^/g, respectively. Doping leads to an increase in the SSA. The powders produced using glycine with 0.1 and 0.4 moles of Si^4+^ possessed specific surface areas of 146 and 97 m^2^/g, respectively. When the fuel was citric acid, doping with 0.1 and 0.4 moles of Si^4+^ changed the specific surface area to 34 and 25 m^2^/g, respectively. The TEM images of the powders are presented in Figure 16. It can be seen that doped particles possessed a much more porous structure, influencing the specific surface area.

Bioactivity, cell viability, bone-like nodule formation ability, biodegradability, and cell attachment studies were conducted using Simulated Body Fluid (SBF) media. For example, to evaluate bioactivity, the so-called dynamic surface tension (SFT) of the SBF medium containing the NPs was measured using the Du Noüy ring method. Based on the SFT results, the relative bioactivity was estimated as follows:

(3) $$\beta =\frac{Final\;SFT-SFT\;of\;SBT}{SFT\;of\;SBF}\cdot 100\%$$

It was shown that the bioactivity of the Si-doped sample with 0.1 mole Si^4+^ synthesized using glycine was 255%, i.e., 2.5 times higher than those of undoped samples. However, the bioactivity of Si-doped samples synthesized using citric acid was less than 100%. The difference in the release of Ca ions explained the effect. The better ionic dissolution in the case of the powders synthesized using glycine might stem from their larger specific surface area, more oxygen defects, and higher zeta potential values. 

The solution combustion synthesis of CaPs doped by Sr^2+^, Fe^2+^, and Ti^4+^ ions was also investigated [90]. It was found that low-dose doping decreased the particle size and significantly enhanced specific surface area, while high-dose doping had the opposite effect (Table 8).

**Figure 17:**
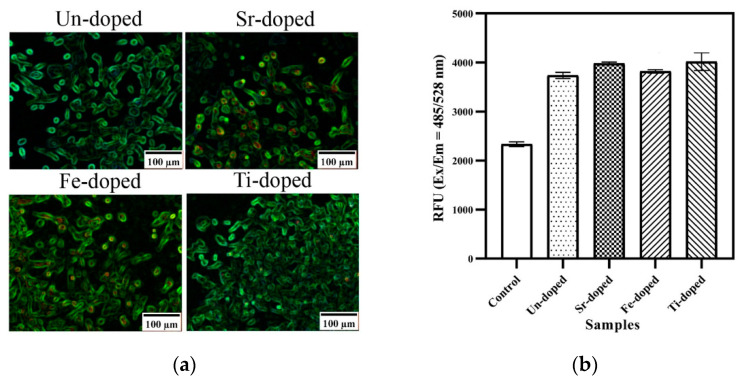
The ROS generation results of doped CaPs with 0.1 mol of theranostic ions. The fluorescent microscope images (**a**) and the results of ROS quantification (**b**). (Reprinted with permission from [90]. Copyright 2021 Elsevier).

The synthesized CaPs showed attractive biomedical properties. Specifically, the in vitro cell-based results indicate that these materials had a significant impact on the generation of reactive oxygen species (ROS). The CaPs alone led to the increase in intracellular ROS generation by about 60%. The doped CaPs with Sr^2+^, Fe^2+^, and Ti^4+^ ions resulted in an even more significant improvement in ROS generation (Figure 17).

In conclusion, the SCS method can be used to synthesize CaPs-based nanoceramics with attractive biomedical properties, and doping can enhance their properties further.

## 4. Challenges and Tasks

The SCS has immense potential for producing materials applicable in diverse biomedical fields. This energy-efficient method allows for control over the phase composition and morphology of particles synthesized. However, significant work remains to be carried out to transform this approach from a promising and attractive technique to a laboratory-scale or industrial technology.

The SCS operates on the principle of self-sustained reactions and differs significantly from conventional methods. All parameters involved in the process—temperature, pressure, time, rate of chemical reactions, etc.—are closely correlated, making trial-and-error optimization challenging. Precise control over the structure and properties of synthesized materials is possible only through a fundamental understanding of the phenomenon.

Combustion synthesis (CS) has over fifty years of history (cf., [91,92]). During this time, a solid theoretical basis has been established, which allows us to predict and explain experimental observations on the synthesis of various materials [64,93,94,95]. Many in situ and operando diagnostics were developed to investigate the phenomenon of self-sustained reactions [91,96]. It was proven that while the synthesis conditions are unique, i.e., extremely high temperatures and the rate of temperature change in the reaction front, during CS, one can use well-acknowledged mechanisms for new phase formation [96]. Unfortunately, analysis of the literature shows that only a few teams in the world are using these priceless accumulations.

Typically, the SCS-related studies involve the preparation of reactive solutions with varying fuel-to-oxidizer ratios, followed by high-temperature treatment using heating sources such as hot plates, furnaces, or microwaves. The properties of the product obtained after the initiation of self-sustained reactions were examined. Such an approach is cost-effective but not suitable for scaling up to a technology level. It is worth noting that the volume combustion synthesis (VCS) mode involving thermal explosion is less controllable than other modes of self-propagating high-temperature synthesis (SHS). Thus, we recommend using the SHS mode for the SCS of materials.

The temperature of the preheating device does not accurately characterize the temperature of new phase formation during SCS. Hence, temperature measurement via thermocouple or pyrometric means is critical in any study of the process to develop reliable technology. Various parameters such as ignition temperature, adiabatic combustion temperature, maximum combustion temperature, and furnace temperature influence the mechanism of the reaction, and therefore the properties of the synthesized material. Thus, their values should be measured and calculated, accounting that the adiabatic combustion temperature depends on the equilibrium phase composition of the products, which can be different for the same system but for various fuel-to-oxidizer ratios, amounts of water, or surrounding atmospheres (see Figure 1). Thus, it is critical to calculating the thermodynamic equilibrium phase composition, followed by an estimation of the adiabatic combustion temperature.

The ignition temperature of the reactive system is not a fixed value and may vary with changes in preheating and heat loss conditions. However, it is often related to the decomposition temperatures of the fuel or oxidizer. Analyzing these values for each system under study enables the control of reaction initiation conditions, essential for controlling the structure formation mechanism.

The combustion mechanism involves gas-phase reactions between gaseous species formed during the decomposition of the fuel and oxidizer. The reaction atmosphere may have a reducing or oxidative nature, which influences the final product’s composition. The control of the gaseous combustible atmosphere is powerful in controlling the product’s phase composition. Studying the combustion mechanism is, therefore, crucial to the SCS process.

One advantage of SCS is the ability to fabricate materials with desired properties directly, without the need for post-combustion calcination. Optimizing the synthesis conditions allows this task to be accomplished for any desired products.

SCS and the ability to form desired organic complexes from reactive solutions permit the fabrication of metastable phases, which are difficult to produce through conventional methods [56,59,74]. Finally, the SCS community should focus on developing SCS-based approaches to produce not only oxides but also metals, alloys, carbides, nitrides, etc. Successful examples of these approaches are available in the literature.

In summary, the SCS is a potent and unique method for fabricating various nanoparticles for biomedical applications. Researchers must pay more attention to the fundamental principles of the phenomenon to design practical and controllable approaches. All diagnostics and techniques for SCS are well known, and using them can help to develop energy-efficient, continuous, and sustainable technology.

## Figures and Tables

**Table 1:** The components of reactive solutions.

Oxidizer	Fuel	Solvent	Atmosphere
Metal nitrate hydrates Me^ν^(NO_3_)_ν_∙nH_2_O; (Me = Fe, Co, Ni, Mn, etc.) —metal valency Metal Oxalates hydrates: Me(C_2_O_4_)∙nH_2_O Ammonium Nitrate: NH_4_(NO_3_) Nitric acid (HNO_3_)	Glycine (CH_4_N_2_O) Citric Acid (C_6_H_8_O_7_) Urea (CH_4_N_2_O) Sucrose (C_12_H_22_O_11_) Glycose D-(+)-C_6_H_12_O_6_ Hydrazine (H_2_N-NH_2_) Carbohydrazide (CH_6_N_4_O) Oxalyhydrazide (C_2_H_6_N_4_O_2_) Metal hydrazinecarboxylates hydrates: (N_2_H_5_Me(N_2_H_3_COO)_3_·H_2_O, where Me = Fe, Co, Ni, Zn) Hexamethylenetetramine: (C_6_H_12_N_4_, HMTA) Acetylacetone (C_5_H_8_O_2_)	Water (H_2_O) Benzene (C_6_H_6_) Ethanol (C_2_H_6_O) Methanol (CH_4_O) Furfuryl alcohol: (C_5_H_6_O_2_) Formaldehyde: (CH_2_O) 2-methoxyethanol (C_3_H_8_O_2_)	Air Nitrogen Oxygen Inert gas: Argon Helios

**Table 2:** Some characteristics of the iron oxide nanoparticles. (Reprinted with permission from [68]. Copyright 2021 Elsevier).

Sample	Heating	Phase Composition wt.%	Crystallite Size, nm	S_BET_ m^2^/g	Average Pore Diameter, nm
P1	Mantle	97% Fe_3_O_4_ + 3% γ-F_2_O_3_	23	42	7.1
P2	Microwave	85% γ-F_2_O_3_ + 11% Fe_3_O_4_ +2% FeO +2%Fe	5	71	5.5

**Table 3:** Some characteristics of colloidal suspensions (adopted from [68]).

Sample	Surfactant	Dispersion Media	Diameter, nm	PDI	Zeta Potential mV
O/W	Oleic acid	Water	91	0.179	−74
O/PBS	Oleic acid	PBS	105	0.326	−47
O/W	Oleic acid + Tween80	Water	105	0.315	−14
O/W	Oleic acid + Tween 80	PBS	79	0.189	−12

**Table 4:** Characteristics of iron oxide powders (adopted from [70]).

Sample	Crystallite Size, nm	S _BET_ m^2^/g	Average Diameter Nm	Saturation Magnetization emu/g	Remnant Magnetization emu/g	Coercively kA/m
Fe_3_O_4_	18	56	21	57.7	4.5	5.2
γ-F_2_O_3_	5	149	8	41.5	0.7	1.0

**Table 5:** Coercive fields (Oe) of BiFeO_3_ samples at different temperatures. (Reprinted with permission from [75] Copyright 2018 American Chemical Society).

Sample	1.8 K	60 K	100 K	200 K	300 K
BiFeO_3_-10	734	330	195	108	80
BiFeO_3_-7.4	1207	484	284	178	172
BiFeO_3_-5.2	1226	692	529	353	288
BiFeO_3_-3.6	1561	827	594	321	198

**Table 6:** Some properties of ZnO particles. (Reprinted with permission from [82]. Copyright 2018 Taylor & Francis).

Sample	Crystallite Size, nm	S _BET_ m^2^/g	BJH, Average Pore Diameter, Å
ZnO(L)	13	19	91
ZnO(A)	11	26	83
ZnO(M)	9	90	56
ZnO(I)	11	38	72

**Table 7:** Semi-quantification results on crystallinity, phases, crystallite, and particle size. (Reprinted with permission from [89]. Copyright 2020 Elsevier).

Fuel	Amount of Si, Mole	Crystallinity %	HA wt.%	βTCP wt.%	Crystallite Size HA, nm	Crystallite Size βTCP, nm	Particle Size, nm
Glycine	0	59	90	10	15.6	42.4	68 ± 6
	0.4	52	86	14	15.6	42.4	60 ± 4
Citric Acid	0	55	74	26	14.2	42.4	84 ± 12
	0.4	50	94	6	14.2	42.4	54 ± 8

**Table 8:** Semi-quantification results on crystallinity, phases, crystallite, and particle size. (Reprinted with permission from [90]. Copyright 2021 Elsevier).

Fuel	Amount Dopant, Mole	Crystallinity %	HA wt.%	βTCP wt.%	Crystallite Size, nm	BET. m^2^/g	Particle Size, nm
Un-doped	0	59	90	10	60 ± 4	37	68 ± 6
Sr-doped	0.1	59	91	9	60 ± 4	79	39 ± 2
	0.5	53	93	7	51 ± 6	23	69 ± 3
Fe-doped	0.1	60	90	10	50 ± 3	65	24 ± 6
	0.5	55	87	13	41 ± 5	23	60 ± 6
Ti-doped	0.1	58	88	12	47 ± 9	106	29 ± 5
	0.5	41	84	16	38 ± 13	66	74 ± 16

## Data Availability

Data sharing is not applicable to this article.
